# The anesthetic management of a child with ohtahara syndrome and severe stridor: a case report

**DOI:** 10.1186/s12887-024-04907-8

**Published:** 2024-07-05

**Authors:** Nidhi Patel, Peymon Madi, Iona Monteiro, Shridevi Pandya Shah

**Affiliations:** Department of Anesthesia and Perioperative Care, Rutgers New Jersey Medical School, 538 E-MSB, 185 South Orange Ave, Newark, NJ 07103 USA

**Keywords:** Ohtahara syndrome, Stridor, Bronchoscopy, General anesthesia

## Abstract

**Background:**

Ohtahara syndrome is a progressive developmental and epileptic encephalopathy that manifests in the early infantile period. This rare condition is characterized by intractable seizures, psychomotor retardation, and poor prognosis. To date, there are a handful of case reports regarding the anesthetic management of children with Ohtahara syndrome. However, limited reports exist of patients with Ohtahara syndrome who present with difficult airways. This report describes our airway findings and general anesthetic management of a pediatric patient with Ohtahara syndrome undergoing diagnostic bronchoscopy for severe inspiratory stridor.

**Case presentation:**

A 14-month-old, 9 kg, male patient with Ohtahara syndrome presented with a year-long history of severe inspiratory stridor and was scheduled for bronchoscopy with lavage. On exam, the patient had noisy breathing, was non-verbal with developmental delay, and had poor head control with significant central hypotonia. The patient was induced with ketamine and general anesthesia was maintained with propofol. Bronchoscopic evaluation was completed uneventfully and revealed a diagnosis of laryngotracheomalacia. The patient’s breathing was maintained spontaneously throughout the procedure and no seizures were noted. In the post anesthesia care unit, the patient’s respiratory and cardiovascular function were stable.

**Conclusions:**

This report documents the unusual finding of severe inspiratory stridor in a 14-month-old child diagnosed with Ohtahara syndrome and our anesthetic management during their diagnostic bronchoscopy. Currently, documentation of complex airway pathology present in patients with Ohtahara syndrome is limited and should be further evaluated. This will assist pediatric anesthesiologists as these patients may require careful preoperative assessment, thoughtful airway management, and surgical alternatives on standby.

## Background

Ohtahara syndrome is an early infantile epileptic encephalopathy that was first described in 1978. Patients with Ohtahara syndrome are typically diagnosed in the first few months of life based on a combination of frequent tonic spasms that are refractory to anticonvulsant therapy and persistent suppression-burst patterns observed on electroencephalogram (EEG) independent of the circadian cycle. [1] This progressive syndrome is associated with structural brain malformations that can evolve into other epileptic encephalopathies such as West syndrome and Lennox-Gastaut syndrome. [2]

Stridor is a monomorphic, high-pitched sound produced by turbulent air flow through a narrowed segment of the upper respiratory tract. It is the most prominent symptom of airway obstruction in infants and can be either acute or chronic. Given that stridor is a symptom and not a diagnosis, appropriate management is only possible once a precise diagnosis has been established [3]. When performed under anesthesia, flexible bronchoscopy has been shown to have increased sensitivity and specificity and is therefore the diagnostic procedure of choice [4]. Laryngomalacia is the most common congenital cause of extrathoracic airway obstruction in infants, and is characterized by inspiratory stridor that is worsened with feeding and laying supine. The disease course typically progresses during infancy and resolves by 12–18 months. However, untreated laryngomalacia carries a significant risk of morbidity [5]. We present our airway findings and general anesthetic management of a pediatric patient with Ohtahara syndrome undergoing diagnostic bronchoscopy for severe inspiratory stridor.

## Case presentation

A 14-month-old, 9 kg male presented with a year-long history of severe stridor and scheduled for bronchoscopy with lavage. The patient was born at 41 weeks of gestation by cesarean section. Following observation of frequent tonic spasms that were refractory to anticonvulsants and an abnormal EEG showing suppression-burst pattern during both sleep and awake states, he was diagnosed with Ohtahara syndrome within the first few days of life. During his neonatal intensive care unit course, the patient was noted to have inspiratory stridor and difficulty with feeding. The patient underwent gastrostomy tube (G-tube) placement at 3 months of age due to persistent poor feeding and suspicion of stridor secondary to laryngomalacia, tracheomalacia, or gastroesophageal reflux disease (GERD). Following discharge, the patient continued to exhibit seizure activity demonstrated by eye twitching and shoulder tremors despite anticonvulsant therapy with levetiracetam, phenobarbital and topiramate. He had regular appointments with a pulmonologist and otolaryngologist for his stridor and respiratory difficulties.

Following the placement of G-tube, the patient was scheduled for bronchoscopic evaluation of the stridor and assessment of need for tracheostomy. The child had failed to reach appropriate developmental milestones. The patient exhibited noisy breathing, poor head control with significant central hypotonia, and was non-verbal.

**Fig. 1:**
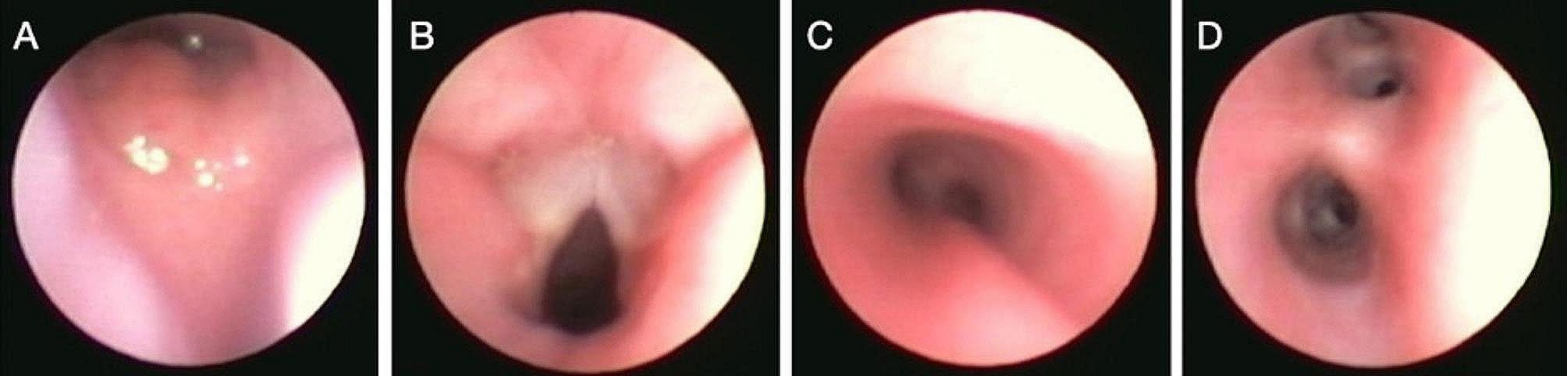
(**A**) Moderately elongated epiglottis consistent with laryngomalacia. (**B**) Vocal cords are symmetric with normal arytenoids. No clefts. (**C**) Mild to moderate tracheomalacia. (**D**) Left and right main bronchus. No fistulas

In the operating room, standard monitors including pulse oximetry, electrocardiogram, and non-invasive blood pressure were applied. A standby ventilator was kept ready along with an otolaryngologist in preparation for emergency tracheostomy. Preoperative vitals revealed a blood pressure of 107/57 mmHg, pulse rate of 155 beats per minute, respiratory rate of 22 breaths per minute, and oxygen saturation of 95%. Preoxygenation was performed and the patient was injected intramuscularly with 0.2 mg of glycopyrrolate and 30 mg of ketamine for induction. Following placement of a 24-gauge intravenous catheter in the right foot, general anesthesia was maintained with intravenous boluses of propofol using a 3 mL syringe with 5 mg given right before the introduction of the bronchoscope. At this time adequate depth of anesthesia was obtained without any observable discomfort upon introducing the bronchoscope into the oropharynx. Thus, a decision was made to proceed without any topicalization. Another 5 mg of intravenous propofol bolus was given right before the bronchoscope was advanced through the vocal cords; a total of 10 mg of propofol was given for the entirety of the case. Spontaneous respiration was maintained throughout the procedure. Bronchoscopy with lavage was completed uneventfully and revealed a final endoscopic diagnosis of laryngotracheomalacia. Bronchoscopy showed mild to moderate tracheomalacia and a moderately elongated epiglottis (Fig. 1). Over the 60-minute procedure, the patient received 3 L of oxygen via nasal cannula and 200 mL of intravenous fluids. He was administered 1 mg of ondansetron shortly before the operation concluded.

In the post anesthesia care unit the patient was tachycardic with a heart rate ranging from 180 to 210 beats per minute. Respiratory and cardiovascular functions were otherwise stable. No seizures were noted during the recovery period. After six hours of observation the patient was discharged with his parents with instruction to follow up in one week. Given the patient’s severe pulmonary pathology, the authors acknowledge that it would be more appropriate for the patient to have been admitted to the pediatric intensive care unit for 24 h to ensure adequate monitoring and to minimize the risk of any associated postoperative cardiopulmonary morbidity. The patient continues to follow up for neurologic and pediatric care at our hospital.

## Discussion and conclusions

Ohtahara syndrome is a rare epileptic syndrome with a poor prognosis. This progressive disease manifests early in life and is diagnosed by persistent seizure activity with suppression-burst pattern on EEG regardless of the circadian cycle. To date, only three case reports have been documented regarding the anesthetic management of a pediatric patient with Ohtahara syndrome [6, 7]. Anesthetic challenges include the reduced ability of patients to cooperate and communicate, the high likelihood of seizure activity, and the potential effects of polypharmacy.

Ohtahara syndrome is known to be associated with structural brain malformations and destructive encephalopathies. However, there is limited evidence in the current literature indicating an association between Ohtahara syndrome and upper airway abnormalities such as laryngomalacia. Laryngomalacia is the most common cause of infantile stridor. It is commonly associated with GERD (65–100%), neurological diseases (20–45%), and less frequently with additional airway lesions, congenital heart disease, and genetic syndromes. Neurologic disease is present in up to 45% of infants with laryngomalacia and includes seizure disorder, hypotonia, and developmental delay [8]. Despite this prevalence, there is only one previously reported case that documents laryngomalacia and Pierre-Robin syndrome in a patient with Ohtahara syndrome [9].

We present an additional case of upper airway abnormalities in a patient with Ohtahara syndrome. While further studies are required as our finding is limited to one patient, we aim to highlight anesthetic considerations in regards to airway management of pediatric patients with Ohtahara syndrome. Upon literature review, there are four main anesthetic techniques when caring for children with difficult airways: general anesthesia with intravenous maintenance, inhalational induction, high frequency jet ventilation and finally sedation with regional anesthesia [10, 11]. This case successfully demonstrates the use of general anesthesia with intravenous maintenance for bronchoscopy with careful consideration of both neurologic and airway implications.

Because Ohtahara syndrome is characterized by tonic spasms refractory to anticonvulsants, there is potential for seizure-like activity to take place perioperatively. Central hypotonia can lead to collapse of the soft tissue structures that maintain a patent airway and affect the swallowing mechanism, leading to airway obstruction and aspiration. Given the diagnosis of stridor and the unknown etiology, we chose to employ an anesthetic management that maintained spontaneous ventilation [12, 13]. This was accomplished via induction with ketamine and maintenance with low-dose propofol.

Propofol was employed due to its anticonvulsant properties with consideration for its potential risk of causing apnea at higher doses. Ketamine decreases the required dose of propofol while also maintaining spontaneous ventilation at higher doses [14]. While intranasal ketamine would perhaps be a better option to consider in the future in order to avoid any additional distress to the patient, this route of administration was not available at the time at our institution. We also used glycopyrrolate to counteract the increased secretions caused by ketamine. Other options to consider include dexmedetomidine and remifentanil use which can reasonably be titrated to effect. However we wanted to ensure an adequate depth of anesthesia in order to avoid any complications such as bronchospasm in this patient with reactive airway features and to facilitate induction in an expeditious manner in order to shorten the overall length of the procedure, hence why we did not use dexmedetomidine. We also wanted to avoid any further respiratory depression and to balance the hemodynamic side effects of propofol with ketamine and thus we decided not to employ remifentanil. The synergistic effect of these ketamine and propofol, along with the maintenance of spontaneous breathing, enabled successful navigation of both neurologic and airway challenges. The procedure was thus uneventful and tolerated well by the patient without any epileptiform activities perioperatively. The authors recognize that the use of ketamine has been associated with slightly prolonged increase in the duration of seizure activity in some studies relating to electroconvulsive therapy, however there is also well documented evidence of seizure termination and shortening of the duration of seizure activity in patients with status epilepticus. One known mechanism is that the NDMA receptor antagonism is able to limit the glutamate mediated excitotoxic activity and thus increase the seizure threshold [15–17]. Nonetheless we acknowledge that the use of agents like midazolam, propofol alone, lidocaine and volatile anesthetics are reasonable options for induction due to the above mentioned concern. Another consideration would be the employment of EEG monitoring intraoperatively to detect any seizure activity during general anesthesia.

The patient has continued to follow up at our hospital since the bronchoscopy documented in this case report. He continues to display seizure-like activity despite trials with various antiepileptic medications. While his stridor has improved, he has experienced respiratory and gastrointestinal complications including obstructive sleep apnea, aspiration, and feeding difficulty. The patient has developed kyphosis and is currently undergoing orthopedic evaluation.

This report documents our unusual airway findings in a pediatric patient with Ohtahara syndrome and our anesthetic management during diagnostic bronchoscopy. We encourage further studies to evaluate the presence of complex airway pathology in children with Ohtahara syndrome. This knowledge may assist pediatric anesthesiologists in making well-informed decisions regarding the airway management of these patients during anesthesia care.

## Data Availability

The data and imaging results are available from the corresponding author on reasonable request.

## Abbreviations

EEG: Electroencephalogram
G-tube: Gastrostomy tube
GERD: Gastroesophageal reflux disease
